# Revealing the impact of teaching methods on anxiety among college students through a bibliometric study

**DOI:** 10.3389/fpsyg.2025.1558313

**Published:** 2025-05-12

**Authors:** Zhongzhu Ai, Dongfeng Yuan, Ruotong Dong, Yun Li, Shanshan Zhou

**Affiliations:** ^1^Faculty of Pharmacy, Hubei University of Chinese Medicine, Wuhan, China; ^2^The First Clinical Medical School, Hubei University of Chinese Medicine, Wuhan, China

**Keywords:** teaching methods, anxiety, college students, bibliometric, trending topics

## Abstract

**Objective:**

Anxiety has become increasingly prevalent among university students, significantly affecting their academic performance. Reforming teaching methods as a potential strategy to alleviate anxiety has garnered growing attention over the years. This study aims to systematically analyze and discuss the impact of teaching method reforms on college student anxiety.

**Methods:**

The Web of Science (WOS) database was used to retrieve and collect relevant literature from 2004 to 2024. Major publication sources, countries, institutions, and authors in this field were identified through the number of publications, citation frequency, and H-index indicators. Data-driven analysis was conducted to explore collaboration patterns, knowledge structures, research hotspots, and trends using VOSviewer software.

**Results:**

After screening, this study included 192 publications from January 2004 to November 2024, revealing several significant findings: (1) The number of publications has gradually increased, peaking in 2022 and maintaining a high level in the following years. (2) The most productive and influential journals are Nurse Education Today and CBE Life Sciences Education, with the USA and the Indiana University System contributing the most. (3) Collaboration network analysis indicates the presence of multiple research groups in the field, but their connections among countries and authors remain relatively limited. (4) Co-citation analysis of journals shows that the field is interdisciplinary, primarily merging psychology, education, and information technology. (5) Keyword analysis identifies two major research hotspots: factors influencing student anxiety in teaching environments (e.g., self-efficacy, loneliness, and performance) and the moderating effects of teaching method reforms (e.g., blended learning, collaborative learning, and experiential learning) on college student anxiety.

**Conclusion:**

This visualization analysis provides an overview of the directions and advancements in research on the impact of teaching method reforms on college students' anxiety. It offers a comprehensive examination of the latest frontiers and trends, contributing theoretical support to educational practices and mental health interventions.

## 1 Introduction

In recent years, anxiety among college students has become a significant issue, attracting widespread attention from both the higher education and mental health sectors globally (Mou et al., 2024). Approximately 31% of college students worldwide experience varying degrees of anxiety symptoms (Auerbach et al., 2018). Anxiety disorders, including generalized anxiety disorder, social anxiety disorder, and panic disorder, significantly affect academic performance and overall wellbeing (Farrer et al., 2016; Sladek and Doane, 2015; Molavi et al., 2018). Academic pressure, social expectations, family environment, and interpersonal relationship challenges are considered key factors contributing to anxiety among college students (Alfaifi and Saleem, 2022; Campbell et al., 2022). With the diversification of stressors among college students, traditional educational models have proven inadequate in addressing these mental health challenges. There is an urgent need to explore effective approaches to alleviate anxiety and promote mental wellbeing.

As educational reforms advance, innovative teaching methods are emerging as a promising means to reduce anxiety among college students. New teaching models, such as blended learning, collaborative learning (CL), and experiential learning, offer students greater opportunities for interaction, engagement, and self-directed learning. These approaches have been shown to enhance students' motivation, boost their confidence, and reduce anxiety (Durak, 2022; Zheng and Zhang, 2020). For example, an experiment involving 259 Chinese university students found that compared to a control group receiving traditional teaching, those who participated in a 3-month experiential learning program showed significant improvements in depression, anxiety, and phobic symptoms (Guo, 2013). However, some studies have pointed out that improperly implemented teaching methods may increase anxiety among students. For example, research found that students engaged in online learning during the pandemic often experienced heightened anxiety (Akcil and Bastas, 2021). Negative experiences in collaborative learning can trigger social anxiety (Huun and Slaven, 2024). While teaching method reforms show promise in alleviating student anxiety, their effectiveness varies depending on implementation, and a unified conclusion is still lacking. Notably, some scholars have reviewed the effects of specific teaching methods, such as experiential learning and cooperative learning, on college student anxiety (Baghaei et al., 2021; Leidl et al., 2020). However, these reviews are limited in scope and do not comprehensively summarize the diverse approaches and outcomes reported in the literature. Therefore, this study employs bibliometric analysis to systematically explore global trends in this field, offering a holistic perspective by quantitatively assessing a wide range of studies and providing insights that complement traditional reviews.

Bibliometric analysis offers a quantifiable method for identifying research trends, hot topics, and core issues in a field. Compared to traditional qualitative reviews, bibliometric methods allow data-driven analyses that reveal research patterns, knowledge structures, collaboration networks, and emerging topics, thus providing theoretical support for the future development of the field (Chen et al., 2024; Yuan et al., 2024). This study aims to systematically review research on teaching reforms and college student anxiety through bibliometric analysis, exploring collaboration networks, research trends, and emerging issues within this field. This will contribute to theoretical support for educational practices and mental health interventions.

## 2 Methods

### 2.1 Study design

In the present study, we conducted a structured literature review on teaching methods and college student anxiety. The methodology relied on a quantitative analysis of the literature in accordance with the PRISMA-2020 guidelines (Page et al., 2021). Relevant studies were identified and assessed from secondary research sources, providing a global and detailed view of current trends, developments, and gaps in the field, establishing the groundwork for a thorough systematic review.

### 2.2 Participants

This analysis focused on college students with anxiety disorders, including college students, undergraduate students, graduate students, and continuing education students. To capture more relevant studies, other anxiety-related issues, including “generalized anxiety disorder,” “social anxiety disorder,” “panic disorder,” “specific phobia,” “separation anxiety disorder,” “obsessive-compulsive disorder,” “post-traumatic stress disorder,” “selective mutism,” “health anxiety,” “hypochondriasis,” and “adjustment disorder with anxiety” were also considered. College students with depression or major depressive disorder and those experiencing academic pressure were excluded from this study.

### 2.3 Interventions

This study examined how teaching methods affect college students' anxiety levels in higher education. The teaching methods included “lecture-based learning,” “inquiry-based learning,” “project-based learning,” “problem-based learning,” “collaborative learning,” “blended learning,” “case-based learning,” “self-directed learning,” “digital learning,” “technology-enhanced learning,” and “experiential learning.” Studies that only explore the impact of teaching methods on college students' academic performance, learning experience, teaching evaluation, and academic pressure, without focusing on their effects on anxiety levels, are not included in this study.

### 2.4 Data sources

The data for this bibliometric analysis were sourced from the Web of Science (WOS) database, a comprehensive and authoritative academic resource that includes a vast collection of peer-reviewed journal articles, conference papers, and other scholarly publications. It is an ideal resource for capturing global research trends in the fields of teaching reform and student anxiety (Powell and Peterson, 2017).

### 2.5 Search strategy

We retrieved relevant studies on teaching methods and student anxiety from the WOS Core Collection database. To construct a comprehensive research dataset, our search focused on three key areas. First, to broadly capture research related to anxiety and other mental health issues, the WOS search string was set as follows: TS = (“anxiety” OR “generalized anxiety disorder” OR “social anxiety disorder” OR “panic disorder” OR “specific phobia” OR “separation anxiety disorder” OR “obsessive-compulsive disorder” OR “post-traumatic stress disorder” OR “selective mutism” OR “health anxiety” or “hypochondriasis” OR “adjustment disorder with anxiety”). Second, to highlight the importance of teaching reform, the WOS search string was set as follows: TS = (“lecture-based learning” OR “inquiry-based learning” OR “project-based learning” OR “problem-based learning” OR “collaborative learning” OR “blended learning” OR “case-based learning” OR “self-directed learning” OR “digital learning” OR “technology-enhanced learning” OR “experiential learning”). Third, to limit the research subjects to college students, the WOS search string was set as follows: TS = (“university student^*^” OR “college student^*^” OR “higher education” OR “undergrad student^*^” OR “master's student^*^” OR “post-secondary education” OR “undergraduate^*^” OR “tertiary education” OR “doctoral student^*^” OR “Ph.D. student^*^” OR “continuing education” OR “continuing learning” OR “vocational education” OR “community college”). In this retrieval process, the Boolean operator OR was used between the keywords of each subject, and the operator AND connected the three themes. Additionally, the asterisk (^*^) was used as a wildcard to include variations of these keywords.

### 2.6 Literature inclusion and exclusion criteria

#### 2.6.1 Inclusion criteria

According to the PICOS criteria (Moher et al., 2009), the inclusion criteria for the study were: (1) Studies published in peer-reviewed journals; only “article” and “clinical trial” types were included. (2) Publication years were limited from January 2004 to November 2024 to ensure the timeliness of the research and its relevance to the current context. (3) The subjects were higher education students with anxiety disorders. (4) The intervention measures of the study involved different teaching methods, such as collaborative learning, online learning, and blended learning. (5) The outcome indicators were changes in anxiety levels among college students after the intervention.

#### 2.6.2 Exclusion criteria

Exclusion criteria were as follows: (1) non-English articles, reviews, editorials, conference abstracts, posters, protocols, and research highlights; (2) duplicate or retracted publications; (3) studies not directly related to college student anxiety or other relevant anxiety issues; and (4) publications that did not address the impact of teaching methods on student anxiety.

### 2.7 Study sections and data extraction

After conducting the initial literature search in the WOS core database using predefined search criteria, all retrieved records underwent multiple rounds of screening to ensure relevance and quality. The retrieved literature was imported into EndNote X9 (Clarivate Analytics) in RIS format, and duplicate studies were removed. Two independent reviewers (Z Ai and D Yuan) screened potential research articles by reviewing titles, abstracts, and full texts, based on predefined inclusion and exclusion criteria. Discrepancies that arose during the screening process were resolved through discussion and consensus by the two reviewers.

### 2.8 Data analysis

Bibliometrics, a quantitative method for analyzing literature, reveals research dynamics, and development trends in specific academic fields. Its core advantage is generating measurable, reproducible, and objective research outcomes. In this study, we systematically examined the development trajectory of research on teaching method reform and student anxiety over the past two decades by analyzing annual publication counts and citation frequencies. Furthermore, we summarized the publication and citation data of the top 10 journals, countries, institutions, and authors to provide a comprehensive analysis of literature distribution and key contributors to academic productivity in this field.

VOSviewer, a widely recognized bibliometric analysis tool, was employed for data visualization. This software analyzes relationships between nodes to illustrate patterns of scientific collaboration and research trends within a specific field (van Eck and Waltman, 2010). In VOSviewer, analysis nodes include countries, authors, journals, and keywords, which can be tailored to the research objectives and data characteristics. In the visualized network, nodes represent analytical units, with node size reflecting their importance within the network. The connections between nodes indicate the strength of their relationships, with thicker lines representing stronger links. Node colors differentiate clusters, with nodes of the same color belonging to the same cluster. In this study, we first mapped the collaboration networks of authors and countries to illustrate academic collaboration patterns in the field. Second, through journal citation analysis, we identified the publishing institutions with significant academic influence in the field and noted their multidisciplinary characteristics. Finally, using keyword co-occurrence analysis, we constructed a thematic map of the field, highlighting the evolution of research topics and emerging issues.

## 3 Results

### 3.1 Study selection and characteristics

**Figure 1:**
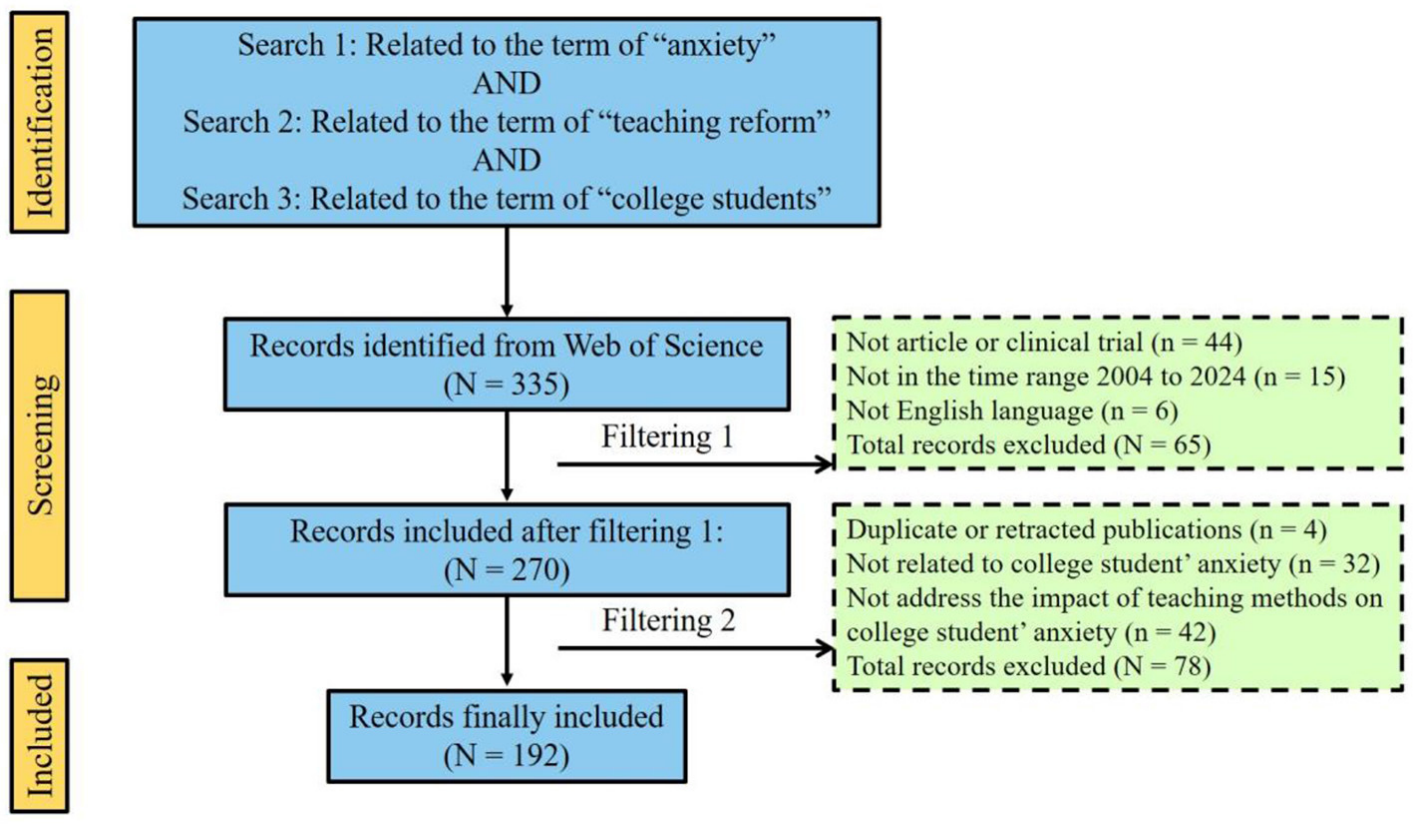
PRISMA flowchart. The literature extraction and screening process in this bibliometric study.

All data used in the study were extracted before December 1, 2024. Initially, 335 publications were retrieved from the WOS database. After the first round of screening, 44 publications that were not categorized as “Article” or “Clinical trial,” 15 publications that were not published in the 2004–2024 range, and six non-English publications were excluded, resulting in a total of 270 publications for subsequent screening. After removing four duplicate or withdrawn publications, the reviewers excluded 32 studies that were not related to college students' anxiety and other mental health issues, and 42 studies not examining the impact of teaching methods on student anxiety were excluded after reviewing the title, abstract, and full text. Ultimately, 192 publications were included in the analysis. The data screening process is detailed in Figure 1.

### 3.2 Publication trends of teaching methods for college students with anxiety

**Figure 2:**
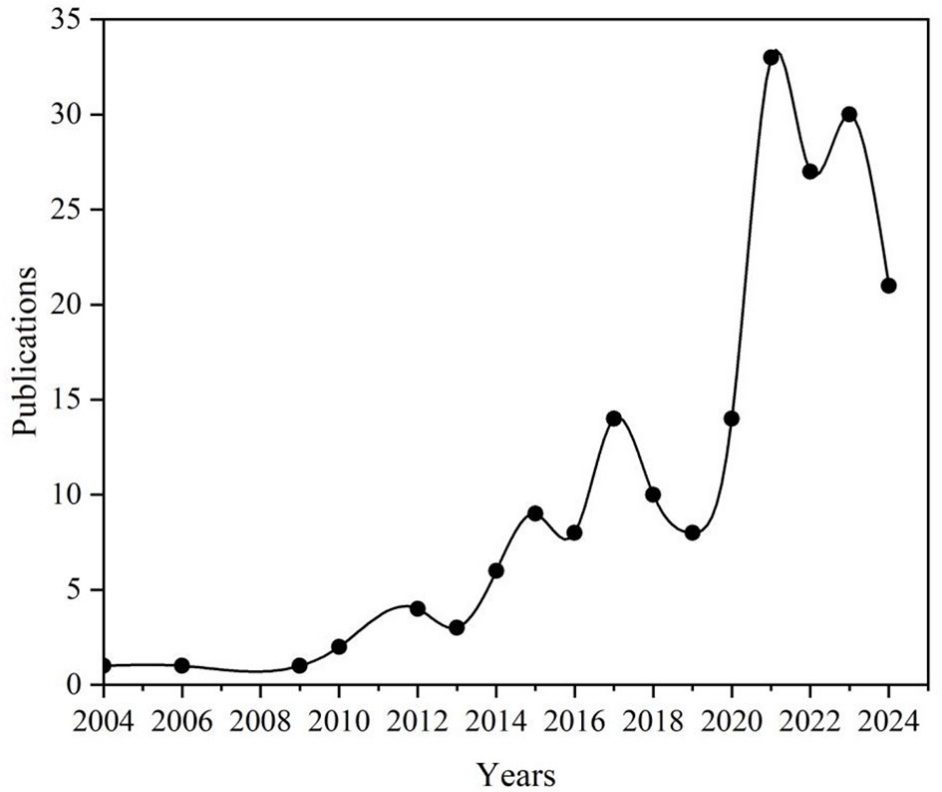
Publications per year on the topic of this study (2004–2024).

Figure 2 illustrates the publication trends in the fields of teaching method reform and college student anxiety, which show several distinct developmental stages. From 2004 to 2013, research output was limited, reflecting the early stage of development in this field. Between 2014 and 2017, the annual number of publications increased moderately, rising from 6 to 14. The subsequent years 2018 and 2019 saw a relatively stable period, with annual publications fluctuating slightly between 8 and 10. Notably, from 2020 to 2023, significant growth occurred, peaking at 33 publications in 2021. While there was a slight downturn in publications in 2024, the output remained high, with 21 publications recorded.

### 3.3 Publications distribution of teaching methods for college students with anxiety

#### 3.3.1 Distribution in major publication source

The top 10 journals by publication number in this field are presented in Table 1. The results indicate that leading journals in this field are primarily concentrated in education and psychology. *Nurse Education Today* and *CBE Life Sciences Education* are the most influential journals, with six and five publications, respectively, and both journals have over 150 total citations. Other significant journals include *Education and Information Technologies* (five publications, 99 total citations), *Education Sciences* (five publications, 120 total citations), and *Frontiers in Education* (four publications, 114 total citations). Their average citations per publication are 19.8, 24, and 28.5, respectively, further demonstrating their standing within the field. Notably, interdisciplinary journals such as *PLoS ONE* (four publications, 124 total citations) and *Sustainability* (four publications, 97 total citations) have also performed well, highlighting the interdisciplinary nature of research in this field. Additionally, medical education journals such as *Nurse Education Today* and *BMC Medical Education* appear in this list.

**Table 1:** Distribution of the literature across the top 10 publication sources.

**Publication sources**	**Publications**	**Count %**	**Citations**	**Average citations**	**H-index**	**JCR (2023)**	**IF (2023)**
Nurse education today	6	3.06	153	25.5	6	Q1	3.6
CBE life sciences education	5	2.55	161	32.2	5	Q1	4.6
Education and information technologies	5	2.55	99	19.8	4	Q1	4.8
Education sciences	5	2.55	120	24	3	Q1	2.5
Frontiers in psychology	5	2.55	35	7	4	Q2	2.6
Frontiers in education	4	2.04	114	28.5	2	Q2	1.9
PLoS ONE	4	2.04	124	31	3	Q1	2.9
Sustainability	4	2.04	97	24.25	4	Q2	3.3
BMC medical education	3	1.53	23	7.67	1	Q1	2.7
Innovations in education and teaching international	3	1.53	47	15.67	3	Q2	1.9

#### 3.3.2 Distribution in major countries/territories

The top 10 countries by publication number in the field are shown in Table 2. The data reveals that the United States and China stand out in research contribution and leadership. The United States leads with 40 publications, accounting for 20.4% of the total, with 611 citations, an average of 15.28 citations per publication, and an H-index of 15, demonstrating significant influence in the field. China follows closely with 33 publications, representing 16.8% of the total, and 370 citations. However, its average citations per publication are lower (11.21), indicating high research output but room for improvement in research quality. Australia ranks third with 19 publications and a high average citation rate (16.74), showcasing strong research quality and international academic influence. Notably, Saudi Arabia, with only eight publications, has a remarkably high average of 23.88 citations per publication, indicating high research quality and citation value. Additionally, the United Kingdom and Germany contributed 18 and nine publications, respectively, with average citation counts of 15.94 and 13.89, reflecting their significant academic standing in the field. In contrast, countries like South Africa and Spain, although producing fewer publications and exhibiting lower average citation counts, show potential as emerging research nations with room for development.

**Table 2:** Distribution of the literature across the top 10 countries/territories.

**Countries/territories**	**Record**	**Count %**	**Citations**	**Average citations**	**H-index**
USA	40	20.408	611	15.28	15
China	33	16.837	370	11.21	11
Australia	19	9.694	318	16.74	10
England	18	9.184	287	15.94	6
Germany	9	4.592	125	13.89	6
Malaysia	9	4.592	139	15.44	6
Saudi Arabia	8	4.082	191	23.88	6
Canada	7	3.571	109	15.57	6
South Africa	7	3.571	39	5.57	3
Spain	5	2.551	30	6	3

#### 3.3.3 Distribution in major institutions

The top 10 institutions by publication number in this field are presented in Table 3. The Indiana University System ranks first with five publications, but its total citation count is 42, with an average citation of only 8.4, indicating high research output but relatively limited citation quality. The Education University of Hong Kong and Nanyang Technological University are tied for second place with four publications each. However, Nanyang Technological University has accumulated 134 citations, with an average citation of 33.5 per publication, demonstrating strong academic influence. Further analysis reveals that the United States dominates in terms of high-impact institutions, such as the State University System of Florida and Maryville College. These institutions not only produce a large number of publications but also have high average citation counts (29.5 and 33.67, respectively). Australian institutions, such as Curtin University and the University of New South Wales Sydney, also perform well in terms of average citation counts, indicating a strong presence in regional research. Notably, the geographic distribution of prominent institutions does not fully align with the national-level analysis. For example, only the Education University of Hong Kong ranked in the top ten among Chinese institutions.

**Table 3:** Distribution of the literature across the top 10 institutions.

**Institution**	**Publications**	**Count %**	**Citations**	**Average citations**	**H-index**	**Countries**
Indiana University System	5	2.55	42	8.4	3	USA
Education University of Hong Kong	4	2.04	57	14.25	3	Hong Kong, China
Nanyang Technological University	4	2.04	134	33.5	3	Singapore
State University System of Florida	4	2.04	118	29.5	4	USA
Curtin University	3	1.53	29	9.67	2	Australia
Manchester Metropolitan University	3	1.53	27	9	2	UK
Maryville College	3	1.53	101	33.67	3	USA
University of London	3	1.53	18	6	2	UK
University of New South Wales Sydney	3	1.53	60	20	2	Australia
University of Tennessee System	3	1.53	101	33.67	3	USA

#### 3.3.4 Distribution of major authors

The top 10 authors by publication number in this field are shown in Table 4. Scholars such as Brigati, Jennifer R., England, Benjamin J., and Schussler, Elisabeth E. stand out in terms of publication volume, total citations, and average citations, demonstrating their significant academic influence in the field. Cooper, Katelyn M. has published only two papers, but her average citation count is 41, indicating that her research is highly recognized in the academic community. Additionally, Zhipeng Zhang and Chwee Beng Lee have accumulated 60 citations, suggesting that their research is also recognized in the international academic community. In terms of geographical distribution, scholars from the United States dominate, with research concentrated in higher education institutions such as the University of Tennessee System and Saint Louis University, reflecting the strong position of the United States in global academic research in this field. Scholars from China, Singapore, Ghana, and Japan, although publishing fewer papers, also show a degree of international academic engagement. Overall, leading authors and research contributions in this field are primarily concentrated in the United States, while scholars from other countries and regions are gradually joining the global academic network with smaller contributions.

**Table 4:** Distribution of the literature across the top 10 authors.

**Name**	**Publications**	**Count %**	**Citations**	**Average citations**	**H-index**	**Institution**	**Countries**
Brandford Bervell	3	1.53	34	11.33	2	University of Cape Coast	Ghana
Brigati Jennifer R	3	1.53	101	33.67	3	University of Tennessee Knoxville	USA
England Benjamin J	3	1.53	101	33.67	3	Saint Louis University	USA
Schussler Elisabeth E	3	1.53	101	33.67	3	University of Tennessee Knoxville	USA
Zhipeng Zhang	3	1.53	60	20	2	Northwestern polytechnic university	China
Cooper Katelyn M	2	1.02	82	41	2	Arizona state university	USA
Xuesong Gao	2	1.02	2	1	1	University of New South Wales Sydney	Australia
Hirakawa Makoto	2	1.02	3	1.5	1	Hiroshima University	Japan
Imura Tomoya	2	1.02	3	1.5	1	Saga University	Japan
Chwee Beng Lee	2	1.02	60	30	2	Nanyang Technology University	Singapore

### 3.4 Analysis of the scientific collaboration network of teaching research on college students with anxiety

**Figure 3:**
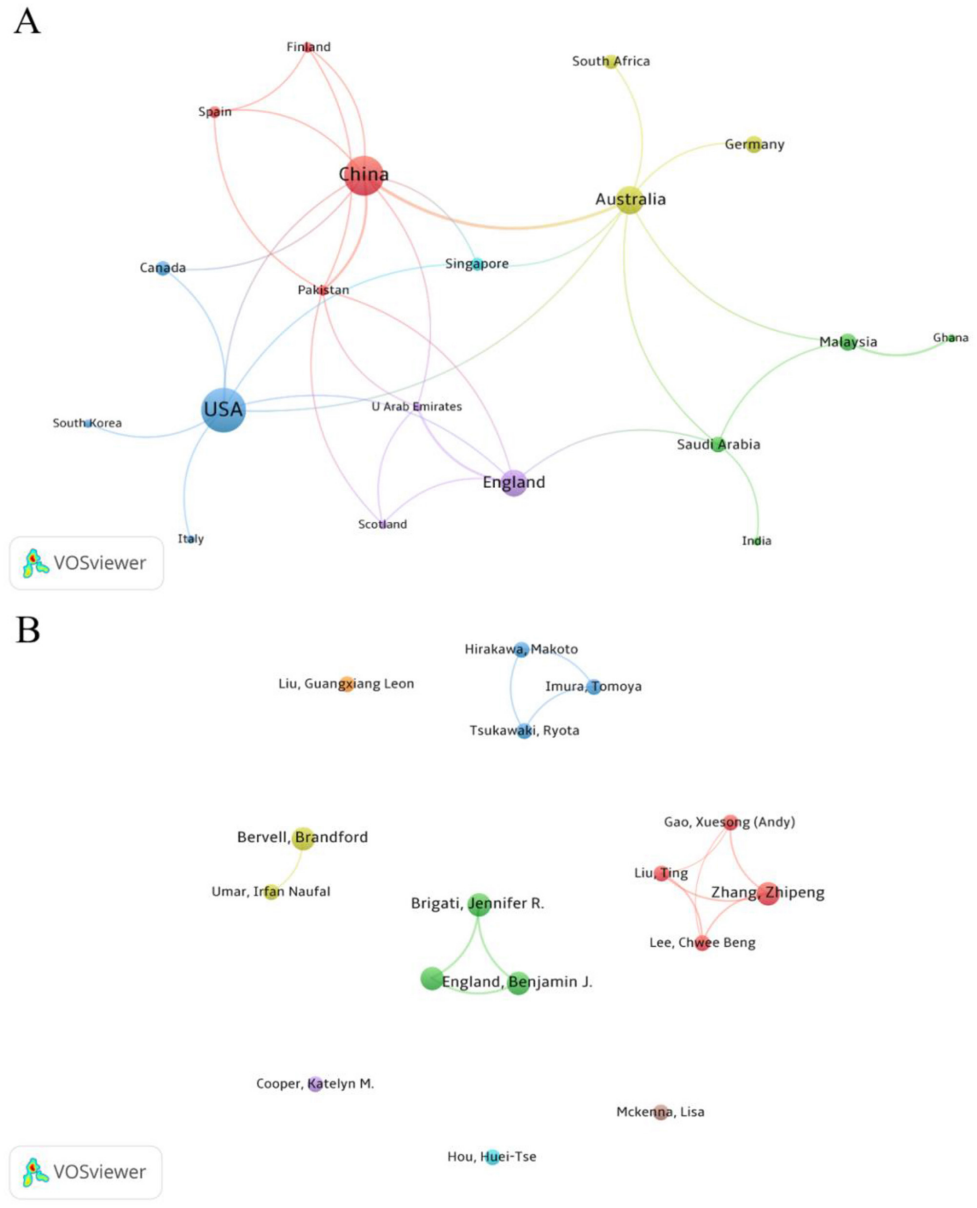
Scientific collaboration networks between countries **(A)** and authors **(B)** in the field of teaching research on college students with anxiety.

This study used VOSviewer software to visually analyze the global scientific collaboration network in the field of teaching reform and college student anxiety from 2004 to 2024. Figure 3A illustrates the structure of the international collaboration network, with lines between nodes representing collaborative relationships between countries or regions and line thickness indicating the degree of cooperation. This analysis included countries that contributed at least three publications from the dataset, resulting in the generation of 21 nodes. The results show that the collaboration network between countries exhibits a significant “strong connection” feature, reflecting the establishment of a robust academic network for international collaboration in this research field. China has established close collaborative relationships with countries such as Spain, Finland, and Pakistan. The United States has formed strong academic links with countries such as Canada, South Korea, and Italy, likely due to shared language backgrounds and similar higher education systems. Overall, collaborative research has driven the globalization of research in teaching methods and student anxiety, enabling countries and regions to enhance the diversity and applicability of research in this field through joint studies and resource sharing. However, it is important to note that the scale of cooperation remains relatively small, and a broad cross-regional and cross-national collaboration network has yet to be established.

Figure 3B illustrates the collaboration network among researchers, where each node represents an individual researcher, and the size of the node reflects the number of publications they have authored. The thickness of the connecting lines between nodes indicates how frequently they co-authored the same article. This analysis focuses on authors who have published at least two articles in the dataset, identifying a total of 16 nodes. The results reveal the presence of multiple collaborative teams in this field. The red cluster, led by Zhang Zhipeng, is the largest collaborative team, consisting of four members and demonstrating close collaboration. Additionally, the blue cluster, led by Hirakawa Makoto, and the green cluster, led by Brigati, Jennifer R., each comprise three researchers and have significantly contributed to the development of this field. However, independent authors represent a large portion of the network, indicating that academic collaboration in the field of “teaching method reform and student anxiety” has not yet formed a broad and unified collaborative network, and the research teams remain somewhat fragmented. This finding suggests that future research should strengthen cross-regional and cross-institutional collaboration to promote systematic and in-depth development of research in this field.

### 3.5 Analysis of co-citation network of teaching methods research on college students with anxiety

**Figure 4:**
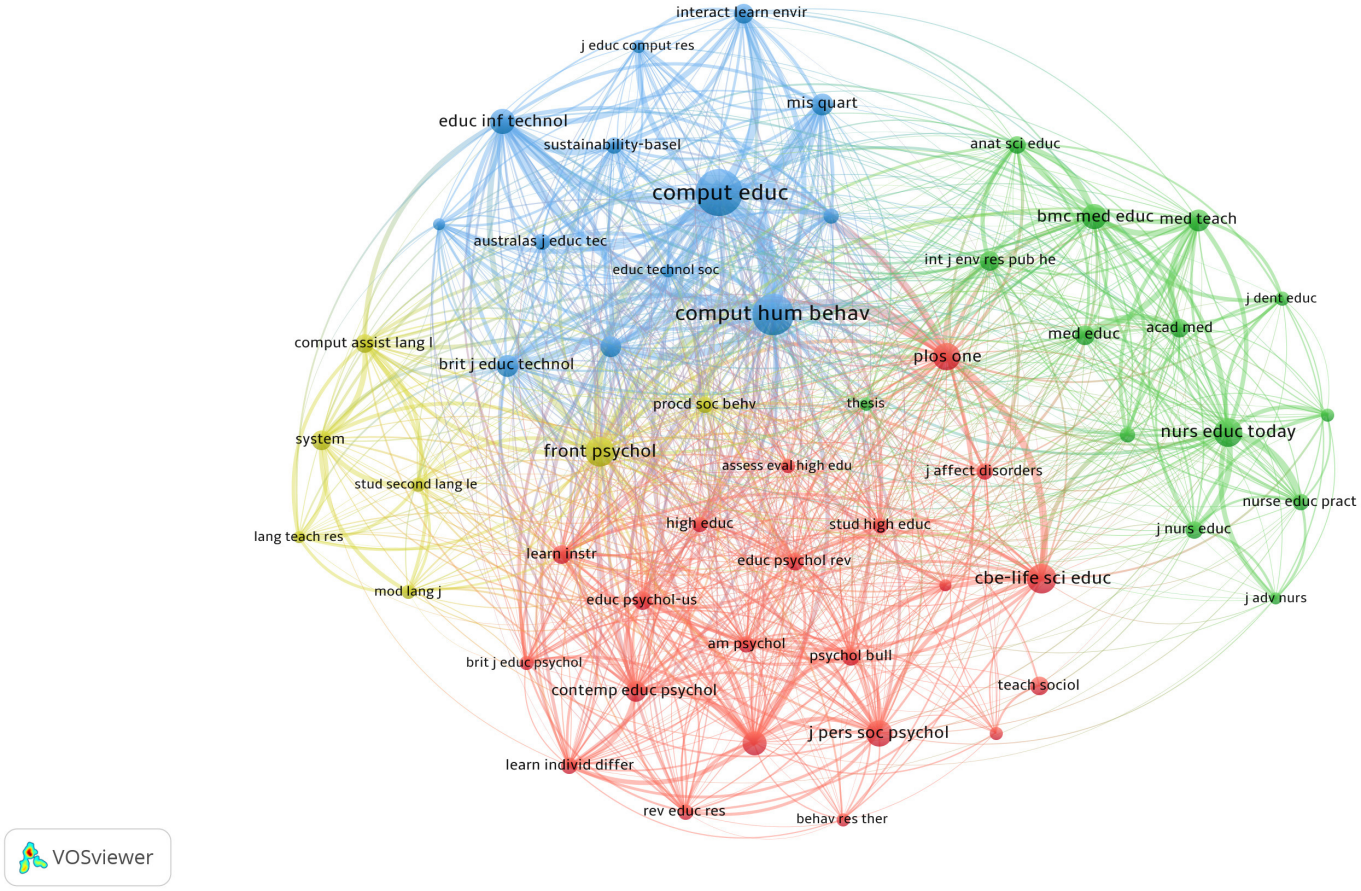
Visualization of the knowledge network of co-citation journals in the field of teaching research on college students with anxiety.

Figure 4 illustrates the co-citation network of journals in the field of teaching reform and college student anxiety. The red cluster primarily includes journals from the fields of psychology and education, such as *Journal of Personality and Social Psychology, Educational Psychology Review*, and *CBE-Life Sciences Education*. These journals mainly focus on psychology, particularly research on personality, social psychology, and educational psychology, emphasizing the significance of a psychological perspective in research within this field. The blue cluster features journals such as *Computers in Human Behavior, Educational Technology & Society*, and *Computers & Education*, which focus on educational technology and behavioral sciences, examining the application of information technology in teaching and its impact on student behavior and mental health. This cluster highlights the connection between digital teaching models and student anxiety. The green cluster includes journals such as *Nurse Education Today, Medical Education*, and *BMC Medical Education*, focusing on medical and nursing education. Research in this cluster focuses on the anxiety issues faced by medical and nursing students, indicating the need for psychological support and optimized teaching methods in high-pressure learning environments. The yellow cluster includes journals such as *Language Teaching Research* and *System*, which primarily cover language education and pedagogical research. This cluster examines the relationship between language teaching and student psychology, particularly anxiety in foreign language learning and innovations in teaching methods. In conclusion, these clusters demonstrate that research in this field spans a wide range of disciplines, including psychology, information technology, medical education, and language studies, highlighting the key role of interdisciplinary collaboration in exploring the relationship between teaching methods and student mental health.

### 3.6 Characteristics of teaching methods on college students with anxiety

Keywords are crucial for extracting and summarizing core terms in research. In this study, VOSviewer was used to analyze the frequency and total link strength (TLS) of keywords. Table 5 presents the frequency of keyword occurrences and TLS data in this field. The five major teaching methods are blended learning, collaborative learning, experiential learning, e-learning, and digital learning. Among these, blended learning ranks first in both frequency of occurrence and TLS, with values of 29 and 27, respectively. The top five keywords related to mental health issues are anxiety, depression, stress, social anxiety, and test anxiety. Among these, anxiety clearly emerges as the main focus of research, highlighting its prominence in the student population and widespread academic attention. Additionally, the primary stressors faced by university students include COVID-19, self-efficacy, loneliness, learning environment, and performance. Notably, COVID-19 has the highest frequency and TLS, with values of 17 and 38, reflecting the profound impact of the pandemic on students' mental health and adaptability in learning.

**Table 5:** The top five keywords in terms of teaching methods, mental health problems, and stressors for college students.

**Category**	**Keywords**	**Frequency**	**Total link strength (TLS)**
Teaching methods	blended learning	29	27
	collaborative learning	15	11
	experiential learning	10	5
	e-learning	10	15
	digital learning	6	7
Mental health problems	anxiety	18	31
	depression	8	17
	stress	5	14
	social anxiety	5	11
	test anxiety	4	3
Stressors for College Students	COVID-19	17	38
	self-efficacy	5	8
	loneliness	3	12
	learning environment	3	4
	performance	3	6

### 3.7 Analysis of research hotspots and trends for teaching methods on college students with anxiety

**Figure 5:**
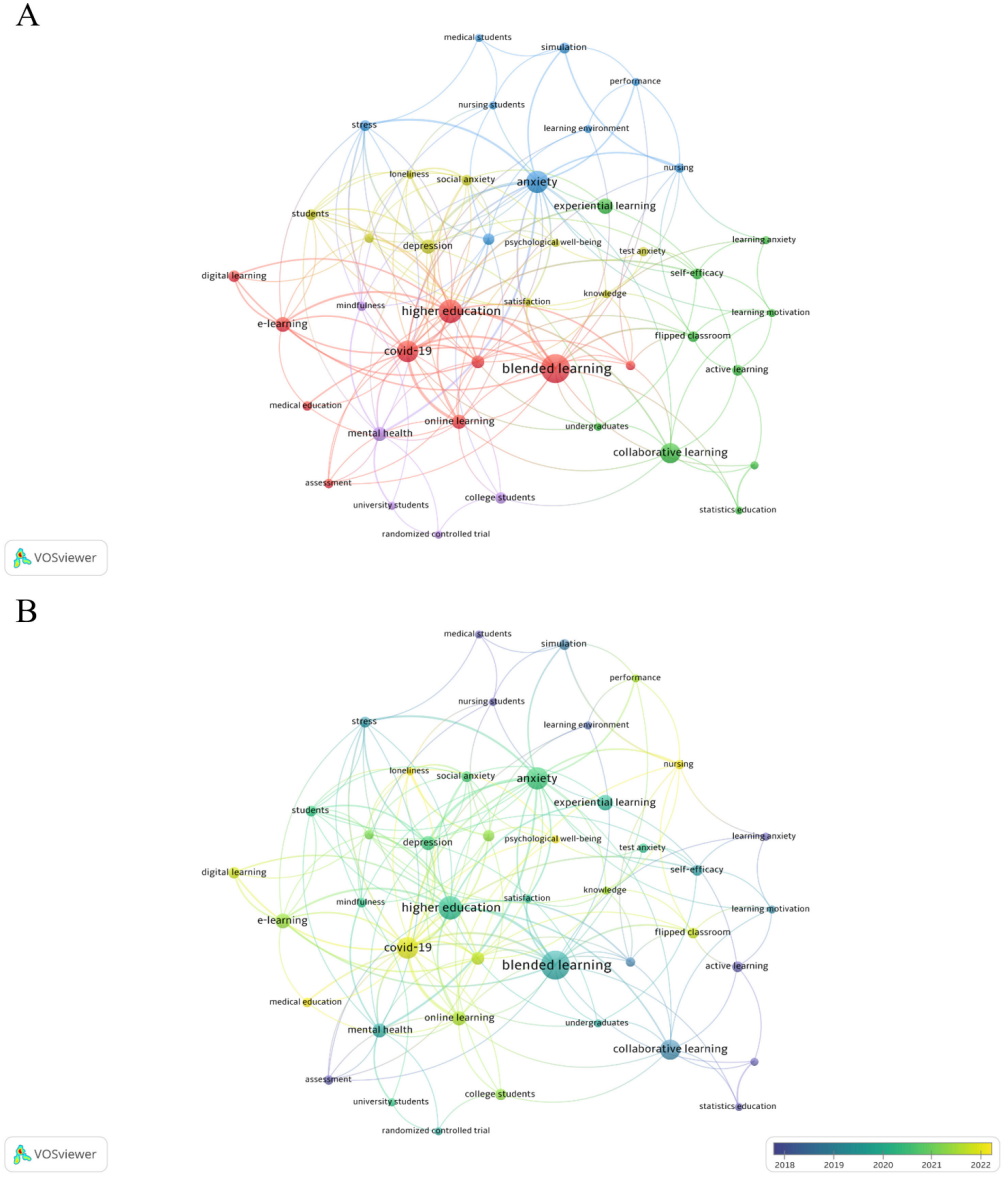
Visualization of the knowledge network for co-occurring keywords in the field of teaching research on college students with anxiety. **(A)** Keyword clustering knowledge maps. **(B)** Keyword timeline knowledge maps from 2004 to 2024.

The keyword co-occurrence network, shown in Figure 5A, reveals the main research hotspots in the study of student anxiety and teaching methods, which can be categorized into five themes. The red cluster focuses on blended and online learning models in higher education, particularly during the COVID-19 pandemic, where the impact of these innovative teaching methods on student mental health has received significant attention. The blue cluster emphasizes factors affecting anxiety, stress, depression, and other mental health issues, particularly among medical and nursing students and their relationship with the learning environment. The green cluster examines the effects of teaching method reforms, such as collaborative learning and flipped classrooms, on promoting student motivation and engagement, while the yellow cluster focuses on the role of experiential learning in enhancing educational satisfaction and alleviating exam anxiety. Furthermore, the purple cluster centers on intervention methods and assessment tools for student mental health, including mindfulness training and randomized controlled trials (RCTs), providing critical support for mental health management.

The visual analysis of keyword trends over time is presented in Figure 5B. Early research in the field of teaching method reform and student anxiety primarily focused on themes such as “stress” and “students,” addressing foundational issues related to academic pressure and mental health among university students. Subsequently, the research focus shifted toward themes like “collaborative learning” and “blended learning,” reflecting scholars' growing interest in how emerging educational models impact student mental health. In recent years, research themes have expanded to include “COVID-19,” “online learning,” “digital learning,” and “loneliness,” highlighting the growing focus on key factors influencing student anxiety in the digital education environment and the increasing recognition of the urgency for mental health support. Furthermore, to comprehensively elucidate the temporal evolution of research hotspots in this field, we employed VOSviewer for keyword co-occurrence visualization analyses for publications across three distinct periods: 2004–2014 (18 publications), 2015–2019 (49 publications), and 2020–2024 (125 publications). These results can be found in the Supplementary Figures S1–S3. Overall, research in this field has evolved from traditional teaching issues to a focus on technology-supported education and personalized mental health interventions.

### 3.8 Assessment of risk of bias

In this bibliometric analysis, we acknowledged that while traditional risk of bias assessments (e.g., using the Cochrane Risk of Bias Tool) are not applicable to our study design, potential biases still exist. For example, high-impact factor journals are believed to publish more innovative and positive research results, indicating possible publication bias. Our analysis shows that the top 10 journals published more than 20% of the total publications (44/196, 22.91%), which may lead to the omission of some negative research results. In addition, we only retrieved and collected papers published in English, which may introduce language bias. Although the WOS Core Collection contains many high-quality journals, this may also mean that some studies from newly established, excellent journals have not been taken into account, leading to database bias.

## 4 Discussion

### 4.1 General status and trends in teaching methods on college students with anxiety

This study uses bibliometric methods to analyze publication trends and distributions, characteristics of the research field, scientific collaboration networks, potential disciplinary structures, and research hotspots to explore the current state and development trends of research on teaching method reform and student anxiety. For data sources, we initially considered four major databases: WOS, PubMed, Scopus, and Google Scholar. After screening and deduplication, we found that the WOS database contains the largest number of high-quality documents, with significantly superior citation accuracy compared to the other databases (Falagas et al., 2008; Wang and Waltman, 2016). Therefore, this study ultimately selected the WOS database as the data source and used visualization analysis methods to provide an intuitive overview of the research status, hotspots, and trends in this field.

Our analysis indicates that over the past two decades, research on teaching method reform and student mental health has steadily increased, peaking in 2021. This growth may be closely linked to the COVID-19 pandemic, which accelerated the adoption of online teaching methods and highlighted the psychological health challenges faced by students during this period (Madigan et al., 2023; Anas et al., 2022). The journal with the highest publication volume is *Nurse Education Today*, while the one with the highest average citation frequency is *CBE Life Sciences Education*. It is noteworthy that two of the top 10 journals are related to medical education, suggesting that students in medical disciplines may face greater mental health challenges and thus warrant more attention (Jiang et al., 2021; Kositanurit et al., 2022). In terms of contributions, both China and the United States have shown significant growth in the number of publications in this field. In the United States, the Indiana University System is the most influential research institution, with Professors Jennifer R. Brigati, Benjamin J. England, and Elisabeth E. Schussler being prominent scholars in terms of publications and academic impact. Keyword analysis reveals that “Blended Learning” is the most prevalent teaching method in current research, while “anxiety” is the primary mental health issue faced by university students, with stress sources often related to “COVID-19.” Co-occurrence of keyword analysis further shows frequent associations between topics like “Blended Learning,” “Collaborative Learning,” and “Digital Education” with anxiety-related terms such as “stress management” and “coping mechanisms,” reflecting broad interest in how teaching reform can alleviate psychological stress in academic environments.

Nevertheless, there is significant room for improvement in multinational and interdisciplinary collaborative research in this field. Network analysis of collaborations shows that research cooperation patterns are influenced by language, cultural background, and geographical location, with concentrations in Europe, the Americas, and Asia. Cooperation in developing countries tends to be more domestic, while contributions from underdeveloped regions remain limited. Currently, international collaboration is a critical pathway for improving research quality, optimizing resource utilization, and expanding academic impact. The research capability gap between developed and underdeveloped countries and its impact on global health research trends can no longer be ignored (Hsiehchen et al., 2015; Akinremi, 2011). Citation analysis of journals indicates that the field has notable interdisciplinary characteristics, with psychology and education as core disciplines. These fields also complement areas such as educational technology, behavioral science, and health-related research. Mabry and colleagues proposed that robust interdisciplinary collaboration can integrate multi-level “causes” in the social-ecological model with the molecular, cellular, and physiological bases of health and disease, driving significant breakthroughs in public health (Mabry et al., 2008). Therefore, future research must strengthen collaboration between developed countries and underdeveloped regions while fostering cooperation among researchers from different disciplines. This will lead to research with greater generalizability and quality, promoting the comprehensive development of the field.

### 4.2 Factors affecting anxiety of college students in teaching environments

The keyword analysis indicates that the main factors influencing mental health issues in the teaching environment, such as anxiety and depression, have become a significant focus in the field. In particular, themes related to “self-efficacy,” “loneliness,” and “performance” were frequently observed. Increasing evidence suggests that low self-efficacy and high levels of loneliness often exacerbate college students' anxiety (Deer et al., 2018; Bryan et al., 2017). At the same time, academic performance, especially when compared to peers or self-expectations, often becomes a source of anxiety for students, particularly in high-pressure learning environments (Abdullah et al., 2021).

#### 4.2.1 Self-efficacy

Self-efficacy is a critical factor influencing university students' academic performance, psychological health, and adaptability. Numerous studies suggest that self-efficacy can effectively alleviate academic-related anxiety. Specifically, students with high self-efficacy experience less anxiety and perform better academically during educational activities. For instance, students with higher levels of social anxiety tend to feel greater anxiety during classroom interactions, such as cold calling (being randomly asked a question), while those with stronger self-efficacy handle these situations more easily and achieve better academic outcomes (Hood et al., 2021). Furthermore, self-efficacy positively impacts academic adaptation in university students. Studies show that students with higher self-efficacy are better equipped to cope with academic pressures and psychological distress during their transition to university life, leading to reduced anxiety (Hen and Goroshit, 2014). A study conducted in Australia found that students with higher self-efficacy exhibited lower levels of anxiety during their first year of university and demonstrated better academic adaptation (Morton et al., 2014). This suggests that enhancing self-efficacy can effectively reduce anxiety caused by changes in the educational environment. Gender also plays a role in how self-efficacy influences anxiety. Research indicates that female students with low self-efficacy tend to experience higher levels of anxiety when facing academic pressures, whereas male students show different responses (Kenney et al., 2015). Educational interventions should consider gender differences and provide personalized support based on students' self-efficacy levels to more effectively alleviate anxiety. Additionally, interventions such as physical exercise or emotional regulation training have been shown to significantly improve students' self-efficacy, thereby indirectly reducing academic procrastination and anxiety behaviors (Ren et al., 2021; Tang et al., 2022). Therefore, enhancing self-efficacy in the teaching environment not only reduces anxiety but also promotes students' academic success and psychological wellbeing.

#### 4.2.2 Loneliness

Loneliness is a common negative emotion among university students, especially during special circumstances such as the COVID-19 pandemic, which has significantly affected their psychological health. Recent studies show that loneliness not only directly correlates with anxiety symptoms but also exacerbates the occurrence and development of anxiety through mediation (Moeller and Seehuus, 2019). For example, a survey conducted during the COVID-19 pandemic found that lockdowns increased students' feelings of loneliness, identifying it as a significant predictor of heightened anxiety (Tasso et al., 2021). Additionally, loneliness not only directly affects anxiety symptoms but also amplifies anxiety responses through its interaction with anxiety. Research indicates that loneliness plays a crucial role in students' emotional support systems and coping strategies, particularly during the pandemic, where these factors may intensify the negative effects of anxiety (Lu et al., 2022; Dodd et al., 2021). Interestingly, the impact of loneliness on anxiety varies by gender. Studies reveal that loneliness has a more pronounced effect on male students' anxiety symptoms, particularly in the context of behaviors such as smartphone addiction, while the relationship between loneliness and anxiety symptoms is weaker for female students, suggesting that gender may moderate the impact of loneliness on anxiety (Gao et al., 2020). Additionally, loneliness is closely linked to academic stress, and their interaction has a significant negative impact on students' mental health. During the transition from high school to university, increased loneliness, driven by changes in the educational environment, exacerbates anxiety and affects academic and social adaptation (Beiter et al., 2015; You and Kang, 2014). In the teaching reform, exploring measures to alleviate loneliness is particularly important for mitigating anxiety in college students. Emotional support, the establishment of healthy social networks, and enhancing self-acceptance may be efficient intervention strategies for alleviating both loneliness and anxiety (Trigueros et al., 2020; So and Fiori, 2022).

#### 4.2.3 Performance

Academic performance is a major source of anxiety for university students, directly impacting their psychological health as it is closely linked to post-graduation opportunities such as employment or further education. Scholars have extensively examined the relationship between academic pressure, performance, and student anxiety in the teaching environment, providing preliminary evidence of how various factors interweave to influence students' emotions and mental states. Numerous studies identify academic performance, academic stress, and uncertainty about career plans as key contributors to student anxiety (Jamieson et al., 2021; Deer et al., 2018). Senior students often face greater academic burdens and uncertainty about future plans, making them more susceptible to higher levels of anxiety (Zhang et al., 2024). As teaching methods evolve, many universities have introduced active learning techniques, such as interactive learning, to enhance student engagement. However, while these methods can reduce anxiety in some cases, research indicates that certain forms of active learning, such as group discussions and random questioning, may actually exacerbate anxiety, especially when students are required to perform in public without adequate preparation (Kalu et al., 2023). This “fear of negative evaluation” is a key source of academic anxiety. Therefore, improving students' mental health, especially through targeted psychological interventions, can effectively enhance academic performance. Notably, supportive social networks are considered a key protective factor for the mental health of university students. Research has shown that students with strong social support cope more effectively with academic pressure and experience lower levels of anxiety (Yamamoto et al., 2023). During the teaching reform process, educators should consider creating a positive social environment to help students build strong social networks. Additionally, incorporating psychological interventions with practical components, such as Cognitive Behavioral Therapy (CBT), has been shown to significantly reduce anxiety and depression symptoms in students (Hassan Kariri and Almubaddel, 2024). These studies suggest that academic performance, teaching methods, mental health, and social support are critical factors influencing student anxiety. Early interventions targeting students' mental health, especially in high-pressure academic environments, can effectively reduce anxiety, improve academic performance, and enhance overall wellbeing. Future research should focus on how to balance academic pressure with mental health to create a more supportive learning environment.

### 4.3 Moderating effect of teaching reform on the anxiety of college students

In our study, keyword analysis revealed that the impact of teaching reforms on college student anxiety is an emerging research focus worth exploring in this field. In particular, four innovative teaching models, namely, blended learning, collaborative learning, experiential learning, and digital learning, have drawn our attention due to their frequent occurrence in the literature. The following section will provide a detailed analysis of the specific effects of these teaching models.

#### 4.3.1 Blended learning

Blended learning, an emerging educational model, combines traditional face-to-face instruction with online learning, enabling students to access course content online while engaging in interactive and practical activities during class (Du et al., 2022; Bazelais and Doleck, 2018). This learning approach is highly favored by college students due to its high interactivity and self-directed nature. During the pandemic, it provided an effective alternative for educational activities (Katal et al., 2023).

Blended learning has a positive effect on alleviating anxiety among college students, likely because it provides more opportunities for self-paced learning, reducing the anxiety associated with time constraints and competitive pressures in traditional classrooms. During the COVID-19 pandemic, many students experienced high levels of anxiety due to difficulties in adapting to online learning and feelings of isolation, with most expressing resistance to this mode of instruction (Baloran, 2020). The emergence of blended learning provides a viable solution to this situation. A study in Ghana involving interviews with 20 undergraduates found that students generally perceived the blended learning model as offering better opportunities for social interaction, which helped reduce academic anxiety (Adarkwah and Huang, 2023). Additionally, the rapid proliferation of mobile technology and social media has provided effective technological support for the implementation of blended learning. A study on integrating cloud learning environments found that using cloud platforms significantly boosted students' motivation and enhanced their professional skills. These benefits can be attributed to the blended learning strategy, which reduced students' anxiety and facilitated access to learning resources and support (Min et al., 2019).

A descriptive study of graduate students at the University of Carabobo revealed that 40% of participants believed blended learning caused stress and anxiety (Malpica and de Villegas, 2014). This suggests that implementing blended learning may increase students' academic burden, particularly among those less familiar with technology or who adapt slowly to new learning models. For instance, a study indicated that students' anxiety when using learning management systems (LMS) is closely related to their adaptation to new technology and their intention to use it (Attuquayefio, 2022). Furthermore, the degree and impact of anxiety vary across students with different personalities. A survey of hotel management students in Hong Kong found that highly extroverted and conscientious students tend to experience lower levels of anxiety, while those with high agreeableness and openness to experience tend to perceive higher levels of anxiety. The blended learning approach is also more effective in improving learning satisfaction among students with low levels of anxiety (Tavitiyaman et al., 2021). This difference underscores the importance of personalized support, particularly in blended learning environments. Therefore, educational institutions should consider students' diverse backgrounds and provide appropriate training and personalized support.

Flipped classrooms, as a form of blended learning, have distinct advantages in promoting active learning and enhancing classroom interaction (Gilboy et al., 2015). In flipped classrooms, students learn foundational content outside of class through videos and course materials, while class time is used for discussions, interaction, and practice (Campillo-Ferrer and Miralles-Martínez, 2021). This teaching method allows students to master content at their own pace, and the high level of class interaction helps alleviate feelings of isolation and helplessness. Furthermore, incorporating gamification elements into flipped classrooms can enhance students' behavioral, cognitive, and motivational engagement, helping to reduce anxiety and silence during the learning process (Ho, 2020). Meanwhile, some studies have highlighted challenges associated with flipped classrooms, including lesson structure, materials used, and delivery infrastructure (Staddon, 2022).

In brief, blended learning, as a flexible teaching model, offers significant benefits in alleviating anxiety among college students. However, attention must be paid to individual differences and technological adaptability during implementation to avoid exacerbating anxiety. In the future, higher education institutions should adjust blended learning strategies based on students' needs and backgrounds. For example, providing additional training support for students unfamiliar with technology and creating more inclusive and supportive classroom environments will help all students adapt and benefit. Additionally, flipped classrooms, as a form of blended learning, should be optimized according to the specific needs of students and the teaching environment.

#### 4.3.2 Collaborative learning

Collaborative learning (CL) is a group-based learning approach designed to promote knowledge sharing and collective intelligence through student interaction and cooperation (Johnson et al., 2007). CL has been shown to enhance students' academic achievement, increase learning motivation, and improve social skills and self-confidence. For example, in language learning, CL improves participants' language comprehension and emotional regulation while reducing feelings of isolation and stress when facing academic challenges (Zhang and Gao, 2024).

Collaborative learning significantly alleviates anxiety among college students. A study involving 58 Iranian English as a foreign language learners (control: 29, experimental: 29) found that students in the experimental group, who received a combined teaching model of collaborative learning, scaffolding, and self-assessment, showed significant improvements in reading comprehension and motivation, along with a notable reduction in reading anxiety (Abdel-Al Ibrahim et al., 2023). This suggests that students benefit from support and feedback in group collaboration, which helps reduce their tension and anxiety. Similarly, the introduction of peer tutors co-teaching with a faculty instructor has been shown to help reduce student anxiety, likely because students find it easier to seek help both inside and outside the classroom, which increases engagement (Gucciardi et al., 2017). Additionally, some studies have found that CL promotes interaction and feedback among students, strengthening their social support networks and alleviating anxiety under academic pressure (Mendo-Lázaro et al., 2022). In CL, students receive timely feedback and support through interaction with instructors, significantly reducing student anxiety while minimizing teachers' time investment, creating a mutually beneficial dynamic for both students and instructors (Hansen, 2024).

While collaboration encourages student participation, interaction, and learning, it may also cause anxiety and stress for those uncomfortable with forced interaction. A cluster-randomized trial involving 1,198 undergraduates showed that pair programming had no significant impact on students' course performance, subject interest, or anxiety (Bowman et al., 2021). Wilkins et al. (2023) analyzed data from 664 higher education students in the U.S. and found that students' technological preparedness, social identity, and cross-cultural communication abilities were closely linked to their intention to participate in collaborative learning. This highlights the importance of considering student characteristics when forming collaborative learning groups. Furthermore, in some online collaborative learning environments, technical factors (such as slow internet speeds outside the classroom and platform user-friendliness) and personal factors (such as anxiety over new technology and lack of investment in the learning experience) may lead students to feel isolated or unable to participate, potentially exacerbating their anxiety (Yusop and Basar, 2017).

Therefore, when implementing collaborative learning, instructors should consider individual student differences, particularly in terms of social skills, technological preparedness, and cultural background. Future research should continue to explore how to optimize collaborative learning strategies across different disciplines and teaching environments to minimize negative effects. Educators and curriculum designers should focus on providing clear role assignments and task objectives in collaborative learning, along with appropriate technological support and guidance, to reduce anxiety caused by external factors during group collaboration.

#### 4.3.3 Experiential learning

Experiential learning, a student-centered approach, emphasizes active participation and gaining knowledge and skills through practical activities (Quibrantar and Ezezika, 2023). Kolb's experiential learning theory emphasizes that learning is a four-stage process: concrete experience, reflective observation, abstract conceptualization, and active experimentation (Lehane, 2020). In higher education, experiential learning offers students with valuable opportunities through simulations, internships, workshops, and other formats, helping them learn from experience and enhance their problem-solving abilities (Wang, 2023). In medical education, this approach has become an indispensable teaching model (Yardley et al., 2012).

An increasing number of studies indicate that experiential learning significantly benefits college students' overall wellbeing, including physical health, mental health, and social adaptability. Studies have found that simulated environments help students face high-pressure situations in low-risk contexts, thus improving their stress resilience. For instance, by simulating emergency situations, students can practice emergency responses in a non-threatening environment. As simulations are repeated, students' anxiety levels decrease, leading to significant improvements in clinical performance (Al-Ghareeb et al., 2019). An interdisciplinary approach in arts education encourages students to express their anxiety and emotions through creative works. This artistic experience not only helps them understand and discuss mental health issues like anxiety but also provides a safe space that enhances their awareness and practical skills related to mental health (Atayero et al., 2021). Additionally, in statistics courses, combining the curriculum with Excel applications, teachers use diverse teaching techniques such as hands-on learning, simulations, and participatory activities, which effectively increase students' interest and engagement in statistics, thereby alleviating anxiety (Park et al., 2022). Elsden et al. explored the role of experiential learning in supporting university students' wellbeing, finding that experiential learning spaces (such as museums, libraries, and gardens) play a positive role in supporting students' mental health, particularly during challenging times like the COVID-19 pandemic (Elsden et al., 2023). Collectively, these studies suggest that well-designed experiential learning approaches can effectively reduce students' anxiety and have a positive impact on their mental health.

While experiential learning has many positive effects, it can also lead to negative outcomes, especially when adequate support is lacking. In some contexts, overly challenging tasks and environmental pressures may increase students' anxiety. For example, in simulated emergency or mental health care scenarios, some students may feel overwhelmed by highly realistic environments, leading to elevated anxiety levels and negatively affecting their clinical performance (Pai, 2016). This phenomenon may cause students to doubt their abilities, which in turn can negatively impact their learning outcomes. Moreover, if the design and implementation of experiential learning lack adequate follow-up support (such as timely feedback and reflection periods), students may not develop the self-awareness and emotional regulation needed for anxiety relief, potentially exacerbating their anxiety (Farooq et al., 2020).

Experiential learning is crucial for medical students, significantly enhancing their clinical skills and psychological resilience. However, excessive challenges and a lack of effective support in the learning environment can exacerbate students' anxiety. To effectively implement experiential learning, educational institutions and instructors should focus on the following aspects: (1) ensuring experiential learning tasks are appropriately challenging without overwhelming students, while providing sufficient preparation time and support; (2) facilitating post-simulation assessments and reflection sessions to help students recognize their progress and areas for improvement, thereby enhancing their self-confidence and emotional regulation skills; (3) designing diverse and flexible learning spaces based on the specific needs of different disciplines, allowing students to experience and reflect in varied environments; (4) using video reviews, instructor guidance, and other methods to help students extract valuable lessons from their experiences and reflect on them in real-life contexts.

#### 4.3.4 Digital learning

Digital learning, also known as online learning, leverages modern information technologies to create a learning environment with new interaction mechanisms and abundant resources (Haleem et al., 2022). Its key features are flexibility and convenience, which eliminate the time and space limitations of traditional education, offering personalized and interactive learning experiences (Müller and Mildenberger, 2021). The core concept is to make learning more autonomous, open, and continuous through the application of information technology (Gellisch et al., 2024).

Instant feedback and flexible learning methods in digital learning allow students to adjust their learning strategies in real-time, enhancing motivation and emotional engagement, which is crucial for alleviating anxiety. Gellisch et al. proposed a three-step digitization approach, and their quantitative analysis showed that compared to traditional methods, students' knowledge gain significantly increased, attention levels significantly improved, enjoyment increased, and anxiety decreased (*p* < 0.001). Additionally, 61.0% of participants expressed a strong preference for the digital course format, and 71.4% acknowledged it was more efficient (Gellisch et al., 2024). Moreover, digital learning offers more personalized learning spaces for students. A study involving 40 undergraduates found that about one-quarter of students preferred digital exercises, believing that the privacy and opportunity for repeated practice were highly beneficial for learning, helping to reduce anxiety associated with face-to-face learning (Lauricella, 2014).

However, more research indicates that when students rely heavily on online learning, some may experience anxiety due to technical issues and a lack of face-to-face interaction. Studies suggest that students may encounter technical issues in digital learning environments, which increase uncertainty in their learning process and, in turn, affect their emotional wellbeing (Novak et al., 2023). Prolonged online learning can also lead to technological fatigue or dependence on electronic devices, further exacerbating anxiety (Ali et al., 2024). Additionally, some studies have explored the factors influencing university students' use of digital learning. A study of higher education students in Indonesia identified factors affecting students' digital learning levels, finding that subjective norms positively influence the use of e-learning, while anxiety has a negative impact. Perceived usefulness (PU) serves as an important mediator between subjective norms (SN), anxiety (AN), and e-learning (EL) (Ansyah, 2022). A larger cross-national study involving 666 university students showed that social anxiety reduced students' interaction in digital learning environments. The level of social anxiety in these environments varied across countries and cultural contexts, with gender differences—female students experienced higher levels of social anxiety than male students (Ifenthaler et al., 2023). Therefore, further research is needed to identify the causes of social anxiety in digital learning environments and develop strategies to minimize its impact.

In conclusion, digital learning plays a dual role in managing anxiety among university students as an important educational method. It can effectively reduce certain anxieties in students by enhancing autonomy and reducing social pressures but may also exacerbate anxiety due to technical issues or a lack of social interaction. Therefore, higher education institutions should consider the potential negative impacts of digital learning and take appropriate countermeasures when implementing it. Firstly, educators should provide technical support to help students address issues encountered during digital learning. Secondly, universities should encourage blended learning, combining online, and offline methods to increase face-to-face interactions, thereby alleviating students' social anxiety. Finally, mental health support should be integral to digital learning, providing online counseling, mental health resources, and peer support to help students effectively manage anxiety during their studies.

### 4.4 Strategies for dealing with anxiety among college students in the teaching environment

#### 4.4.1 Enhance technical support

With the continuous development of information technology, an increasing number of technology-supported learning models are being adopted in university education. Compared to traditional teaching methods, technology-driven reforms, such as distance education and blended learning, allow students to more purposefully acquire knowledge and interact more purposefully through online platforms and technological tools. This largely depends on students' self-directed learning (Baragash and Al-Samarraie, 2018). In this environment, technology plays a crucial role. For example, online learning platforms (such as MOOCs and LMS) and digital education tools (such as learning management systems, online testing, and assignment systems) provide students flexible and personalized learning experiences (Haleem et al., 2022). Virtual learning assistants and intelligent chatbots, utilizing natural language processing technologies, offer real-time question-answering services. Particularly in remote learning and online education settings, these tools effectively help students resolve problems, alleviating anxiety (Okonkwo and Ade-Ibijola, 2021). Moreover, the use of virtual reality (VR) and augmented reality (AR) technologies enhances teaching models. Through virtual scenario simulations, students can practice social skills, manage stress, or engage in relaxation training in a risk-free environment, thereby improving their psychological wellbeing (Al-Ansi et al., 2023).

At the institutional level, big data analytics and learning behavior prediction technologies offer educational platforms to monitor students' learning progress, emotional fluctuations, and learning difficulties in real time (Bai et al., 2021). Through data analysis, educational platforms can adjust learning plans based on students' needs and provide personalized support. Additionally, many universities have implemented online psychological counseling platforms to provide convenient mental health services to students. These platforms typically offer consultations via video, voice, or text, helping students manage learning anxiety, emotional stress, and social phobias (Oti and Pitt, 2021). However, some studies indicate that students' satisfaction with learning management systems (LMS) is significantly influenced by information system quality, computer self-efficacy, and service quality (Ghazal et al., 2018). If universities can optimize the design of technological platforms, reduce students' technology-related anxiety, and enhance their user experience, it will be crucial for reducing anxiety in the learning process. Future research could further explore how to optimize technology support services, particularly in providing more personalized and targeted support, to promote more positive and effective learning experiences for students.

#### 4.4.2 Enhance incentive mechanism

Incentive mechanisms are classified into two types: extrinsic and intrinsic motivation. Extrinsic motivation includes material rewards such as scholarships, course grades, and academic honors, which can directly stimulate students' enthusiasm. Intrinsic motivation, however, stems from students' pursuit of self-actualization and satisfaction with the learning process, reflecting a deeper psychological drive (Jovanovic and Matejevic, 2014). Both types interact to drive students' engagement and performance in learning, thereby alleviating the negative effects of academic pressure and anxiety. The effectiveness of these mechanisms is closely related to students' emotional and psychological states, especially in terms of learning motivation and anxiety management. Vroom's expectancy theory emphasizes that students' expectations of rewards through effort can trigger intrinsic motivation and enhance their engagement in learning (Stahl and Harrell, 1981). In contrast, Deci and Ryan's self-determination theory posits that intrinsic motivation and autonomy play crucial roles in incentive mechanisms, especially when students feel a sense of self-efficacy, which significantly reduces their anxiety and helplessness (Taylor et al., 2014). Therefore, educators should improve the reward system within the teaching environment to enhance students' sense of self-efficacy and achievement, encouraging higher confidence and a more positive attitude toward academic challenges. For example, a study on blended learning modes found that offering academic scholarships, honors, and timely positive feedback helped students feel recognized, reducing anxiety from academic pressure and uncertainty (López-Pérez et al., 2011). Furthermore, neuroscientists argue that the pursuit of rewards motivates daily human behavior. The brain evaluates incoming information, such as the reward value of stimuli, before responding (Hidi, 2016). They suggest that while early-life behaviors are driven by immediate rewards such as the satisfaction of basic needs (e.g., food), long-term rewards, such as a successful career, become more important later in life (Anderson et al., 2011).

These studies indicate that enhancing incentive mechanisms in educational reforms can significantly benefit students by improving their motivation and emotional experiences, such as a sense of achievement and control. Future research could explore more personalized and innovative incentive systems to further enhance their regulatory effect on student anxiety. For example, implementing customized reward systems designed to individual student interests and needs could avoid a one-size-fits-all approach. Additionally, non-material incentives, such as psychological support, mentor feedback, and personalized learning guidance, warrant further investigation.

#### 4.4.3 Create a relaxed and active classroom atmosphere

Educational research shows that classroom atmosphere significantly impacts students' mental health. A relaxed and positive classroom environment alleviates student stress, enhancing their sense of engagement, and belonging. Key components of classroom atmosphere include teacher-student interactions, classroom management strategies, and course design (Barrett et al., 2015). A positive classroom atmosphere can stimulate students' interest in learning and lessen their fear of making mistakes. Teacher-student interaction, open discussion spaces, and supportive feedback are crucial for alleviating student anxiety in the classroom (Gabryś-Barker, 2016). According to Bandura's social cognitive theory, students' self-efficacy is central to overcoming learning challenges and anxiety. Positive feedback and support from teachers can enhance students' self-confidence, thereby motivating them to engage in learning (Stajkovic and Luthans, 1998). Vygotsky's sociocultural theory suggests that learning occurs through social interaction, and the interactions and cooperation in the classroom can help students emotionally adapt to learning challenges (Mercer and Howe, 2012). For example, in collaborative learning models, teachers guide group discussions, and collective activities that help students develop emotional awareness and regulation skills, thereby providing positive support for reducing anxiety (Järvenoja et al., 2020). The flipped classroom model exemplifies this approach, where students engage in personalized learning outside of class, and classroom time is dedicated to group discussions and interaction (Nouri, 2016). Additionally, digital learning platforms offer students more freedom of expression, helping them avoid the tension, and embarrassment that can arise in face-to-face classroom settings, thereby reducing anxiety (Benta et al., 2014).

Therefore, school administrators and educators can alleviate student anxiety by adopting effective teaching methods and technological tools that enhance student engagement, promote interactions and emotional regulation between students and teachers, and reduce stress during the learning process. Future research could further explore the design of classroom atmosphere, such as how to adjust classroom interaction patterns based on students' interests and needs or how to foster psychological safety through classroom culture development. Additionally, as technology advances, the atmosphere of virtual learning environments has become a growing area of research, and it is crucial to explore how to create a more positive and relaxed learning atmosphere in online or blended learning settings.

### 4.5 Strengths and limitations

#### 4.5.1 Strengths

This study draws data from the authoritative WOS database, which provides comprehensive coverage of research on anxiety, diverse teaching methods, and various student populations. The selected literature reflects, to some extent, global developments in the fields of teaching method reform and student anxiety. By employing a range of bibliometric analysis methods, this study presents the research trajectory in this domain, offering a comprehensive view of the distribution of academic productivity. It visually reveals patterns of academic collaboration, disciplinary structures, and research hotspots. This study contributes to a multifaceted understanding of the complex relationship between teaching method reform and student anxiety, providing valuable insights for related researchers. It represents the first comprehensive and systematic bibliometric analysis in this field, exploring the interactions between teaching method reform and student anxiety. It provides a more holistic approach to address issues associated with teaching reform and student anxiety.

#### 4.5.2 Limitations

However, this study has some limitations. First, its reliance on the WOS database may introduce bias in the findings, as other databases such as PubMed, Scopus, and Google Scholar may contain relevant studies not indexed by WOS, potentially offering unique insights in specific areas. Second, although the search terms used in this study were broad, some emerging or niche teaching methods and anxiety-related concepts may have been overlooked. As the fields of education and psychology continue to evolve, new teaching paradigms and factors influencing anxiety are constantly emerging, which may not have been captured by the search terms used in this study. This could affect the understanding of current trends and important research directions in the field. Third, the methodology used in this study relies on quantitative indicators like citation counts, which may overlook essential factors such as originality and practical value of the research. Consequently, this approach may not fully reflect the latest dynamics of teaching practices and student anxiety. Nevertheless, we believe the bibliometric methods used in this study provide valuable insights into the development and current state of the field, as well as the challenges hindering progress, inspiring further research in this area.

## 5 Conclusion

In conclusion, this study reveals trends in the development of the field over the past two decades, providing a comprehensive and systematic analysis of the relationship between teaching reforms and student anxiety. It offers valuable insights and references for further research in this area. First, over the past 20 years, an increasing number of researchers have focused on the relationship between teaching reforms and student anxiety. The continued research interest in this field in recent years suggests that it has not yet reached maturity and will continue to evolve in the future. Second, given the limited collaborative research and the interdisciplinary nature of the field, it is essential to break down barriers between research institutions and foster cooperation among researchers from different regions. Thirdly, through keyword analysis, we explored factors influencing student anxiety, such as self-efficacy, loneliness, and academic performance, and discussed the moderating effect of teaching reform on anxiety among college students. We also proposed strategies for addressing anxiety, such as strengthening technological support, implementing incentive mechanisms, and creating a positive classroom atmosphere. Despite some unavoidable limitations in this study, our research highlights important issues in the field of teaching reforms and college students' anxiety, providing a theoretical foundation and direction for future research and educational practice.

Overall, teaching reforms contribute to reducing student anxiety, though their effectiveness is influenced by multiple factors such as individual differences, instructional design, and technological support. In the future, higher education institutions should combine various teaching methods to create a supportive learning environment that addresses both academic and psychological needs. Teachers should prioritize personalized support and emotional care to help students manage anxiety and improve learning outcomes. In summary, our work will help educators and psychologists in understanding the development and practical significance of teaching method reforms in addressing student mental health issues and contribute to the formulation of more scientifically grounded and effective intervention strategies.

## Data Availability

The raw data supporting the conclusions of this article will be made available by the authors, without undue reservation.
