# Supplementary material for: Revealing the impact of teaching methods on anxiety among college students through a bibliometric study
Source: Front Psychol. 2025 May 12;16:1558313. doi: 10.3389/fpsyg.2025.1558313 (PMC12104304; doi:10.3389/fpsyg.2025.1558313)
Supplement: Supplementary file 1 [file Supplementary_file_1.docx]

**Supplemental material for:**

Revealing the impact of teaching methods in anxiety among college students through a bibliometric study


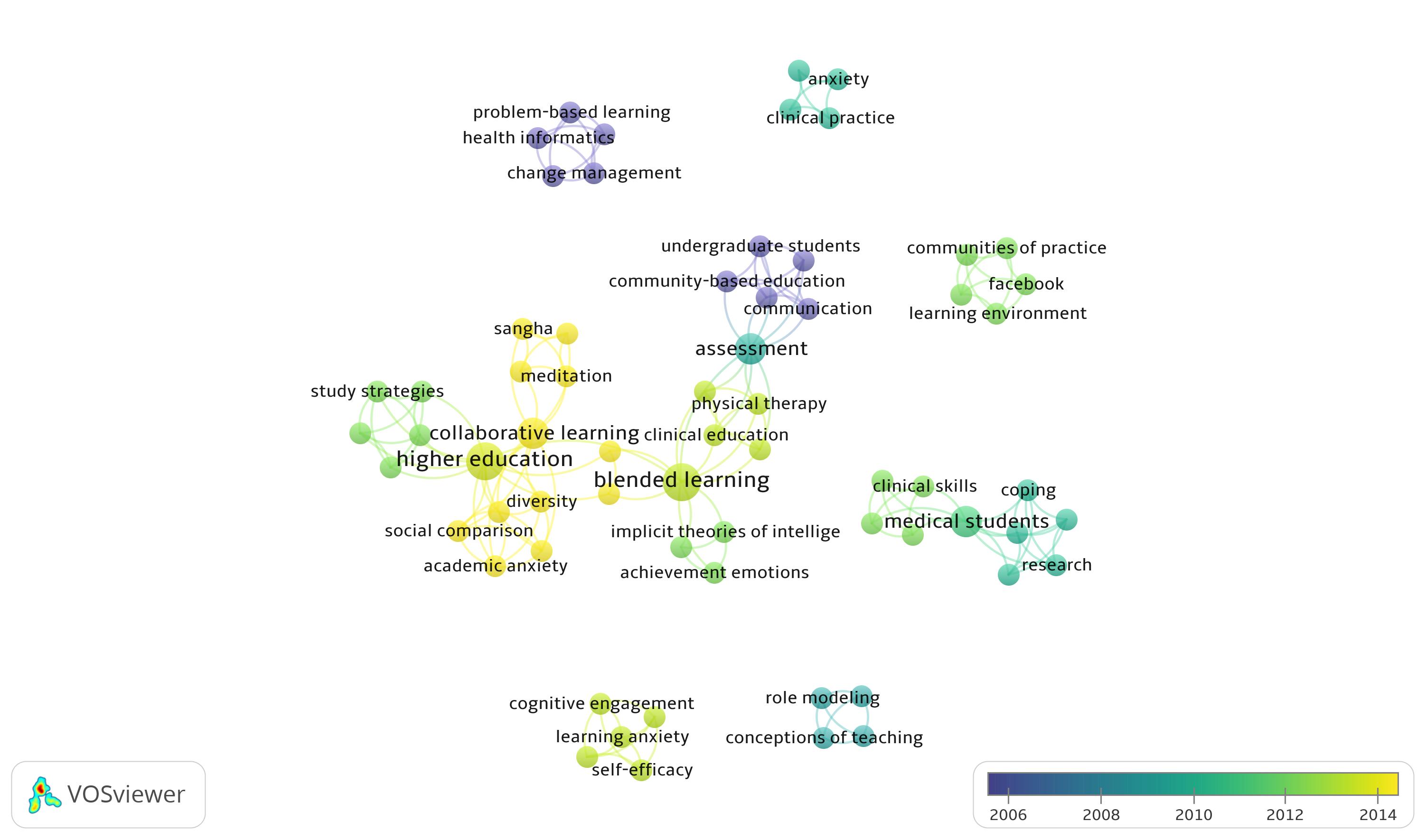


Figure S1 Keyword timeline knowledge maps in the field of teaching methods and college students in anxiety from 2004 to 2014. Keywords with a frequency ≥1 were included, resulting in the inclusion of 65 keywords.


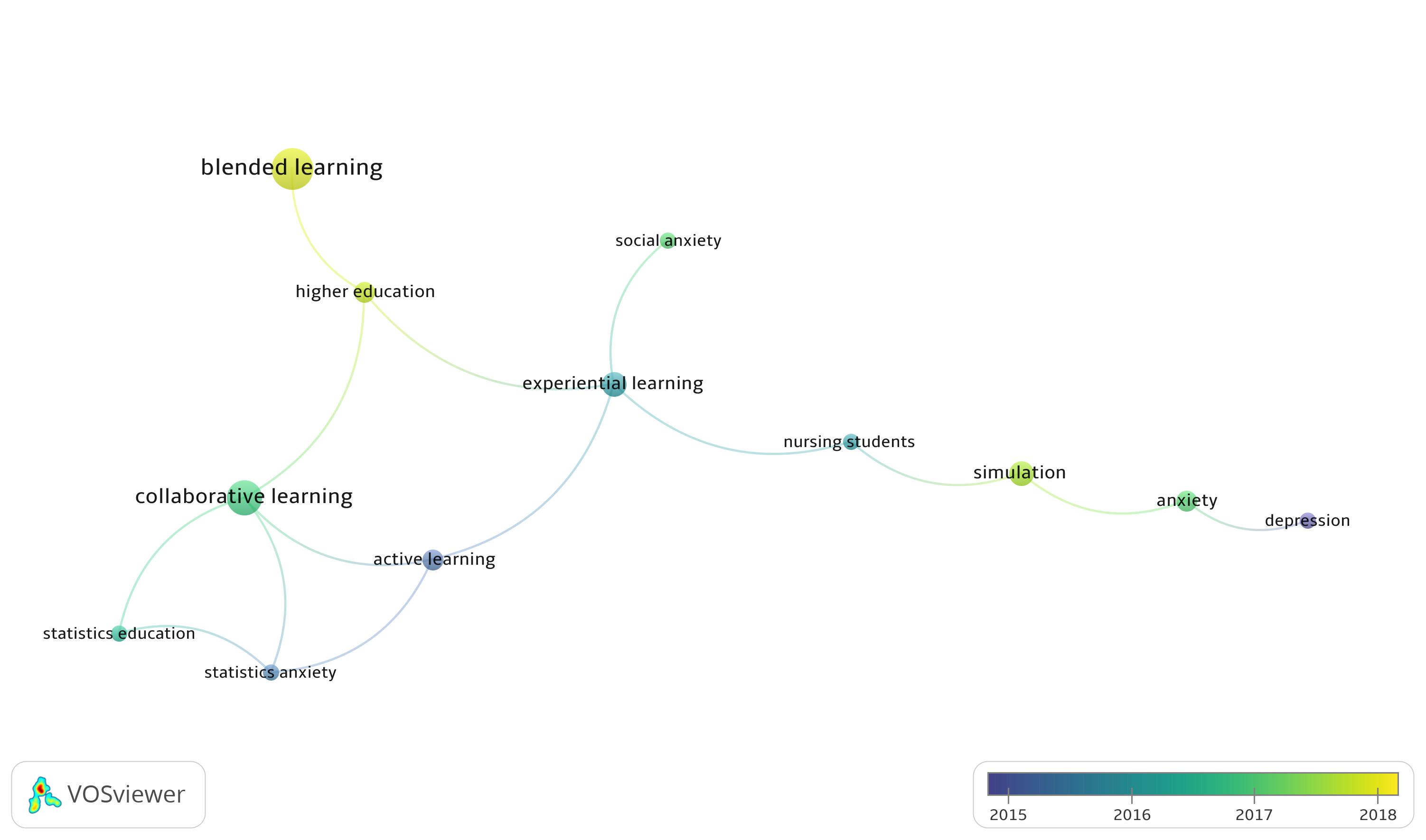


Figure S2 Keyword timeline knowledge maps in the field of teaching methods and college students in anxiety from 20015 to 2019. Keywords with a frequency≥2 were included, resulting in the inclusion of 14 keywords among 181 keywords


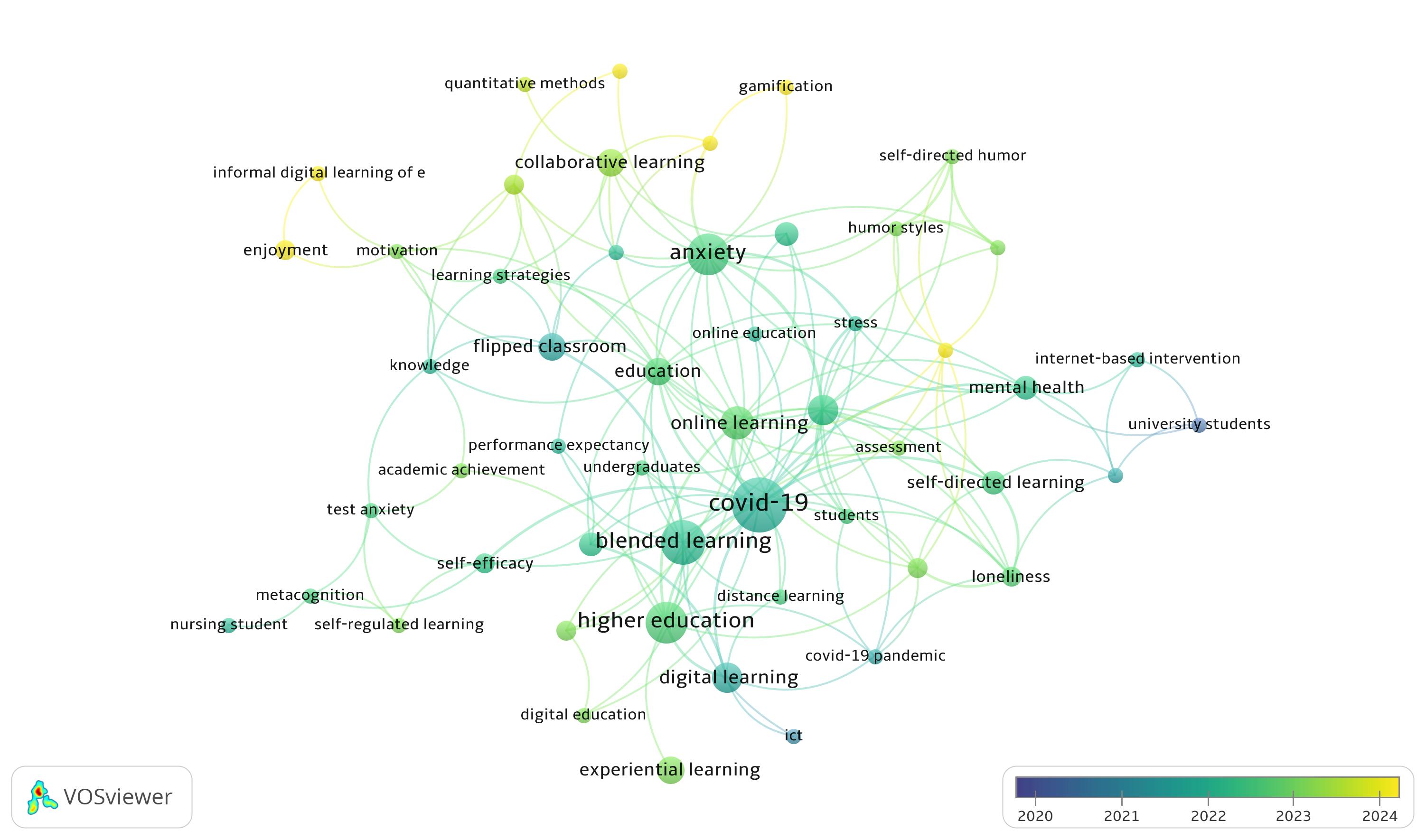


Figure S3 Keyword timeline knowledge maps in the field of teaching methods and college students in anxiety from 20020 to 2024. Keywords with a frequency ≥2 were included, resulting in the inclusion of 52 keywords among 386 keywords.
